# Enhanced Oxidative Damage and Nrf2 Downregulation Contribute to the Aggravation of Periodontitis by Diabetes Mellitus

**DOI:** 10.1155/2018/9421019

**Published:** 2018-12-02

**Authors:** Xumin Li, Xiaoyu Sun, Xiaorong Zhang, Yixin Mao, Yinghui Ji, Lixi Shi, Wenjin Cai, Panpan Wang, Gang Wu, Xueqi Gan, Shengbin Huang

**Affiliations:** ^1^Department of Prosthodontics, School and Hospital of Stomatology, Wenzhou Medical University, Wenzhou, China; ^2^Department of Oral Implantology and Prosthetic Dentistry, Academic Centre for Dentistry Amsterdam (ACTA), University of Amsterdam and Vrije Universiteit Amsterdam, Amsterdam 1081 LA, Netherlands; ^3^Department of Periodontology, School and Hospital of Stomatology, Wenzhou Medical University, Wenzhou, China; ^4^Institute of Stomatology, School and Hospital of Stomatology, Wenzhou Medical University, Wenzhou, China; ^5^Department of Periodontology, Guanghua School of Stomatology, Sun Yat-sen University, Guangzhou, China; ^6^State Key Laboratory of Oral Disease, National Clinical Research Center for Oral Diseases, West China Hospital of Stomatology, Sichuan University, Chengdu, China

## Abstract

Diabetes mellitus is a well-recognized risk factor for periodontitis. The goal of the present study was to elucidate whether oxidative stress and nuclear factor erythroid 2-related factor 2 (Nrf2) participate in the aggravation of periodontitis by diabetes. For this purpose, we assigned Wistar rats to control, periodontitis, diabetes, and diabetic periodontitis groups. Two weeks after induction of diabetes by streptozotocin, periodontitis was induced by ligation. Two weeks later, periodontal tissues and blood were harvested and analyzed by stereomicroscopy, immunohistochemistry, and real-time polymerase chain reaction. We found that ligation induced more severe bone loss and periodontal cell apoptosis in diabetic rats than in normal rats (*p* < 0.05). Compared with the control group, periodontitis significantly enhanced local oxidative damage (elevated expression of 3-nitrotyrosine, 4-hydroxy-2-nonenal, and 8-hydroxy-deoxyguanosine), whereas diabetes significantly increased systemic oxidative damage and suppressed antioxidant capacity (increased malondialdehyde expression and decreased superoxide dismutase activity) (*p* < 0.05). Simultaneous periodontitis and diabetes synergistically aggravated both local and systemic oxidative damage (*p* < 0.05); this finding was strongly correlated with the more severe periodontal destruction in diabetic periodontitis. Furthermore, gene and protein expression of Nrf2 was significantly downregulated in diabetic periodontitis (*p* < 0.05). Multiple regression analysis indicated that the reduced Nrf2 expression was strongly correlated with the aggravated periodontal destruction and oxidative damage in diabetic periodontitis. We conclude that enhanced local and systemic oxidative damage and Nrf2 downregulation contribute to the development and progression of diabetic periodontitis.

## 1. Introduction

Periodontitis, a highly prevalent inflammatory disorder, is characterized by the gradual destruction of the teeth's supportive tissues, eventually leading to tooth loss [1]. Diabetes mellitus (DM), a metabolic disease characterized by hyperglycemia, is a well-recognized risk factor for periodontitis. Epidemiological studies have demonstrated that the prevalence and severity of periodontitis in people with DM are markedly higher than those in nondiabetic subjects. Periodontitis has even been proposed to be the sixth complication of DM [2]. To date, the mechanisms accounting for the aggravation of periodontitis by DM are not completely clarified.

Oxidative stress has been identified as a key pathogenic factor of diabetic periodontitis (DP) [3]. Oxidative stress is characterized by overproduction of reactive oxygen species (ROS) and a relative deficiency of antioxidants [4]. However, it remains challenging to directly detect ROS because of their high reactivity and short half-lives in biological samples. Therefore, the deleterious products resulting from ROS activities are commonly used to evaluate the consequences of oxidative stress-related diseases. 3-Nitrotyrosine (3-NT), 4-hydroxy-2-nonenal (4-HNE), and 8-hydroxy-deoxyguanosine (8-OHdG) represent the most widely accepted assays for assessing oxidative protein damage, lipid peroxidation, and oxidative DNA damage, respectively. However, thus far, only one study has provided limited information on the alteration of local 3-NT level in DP [5]. Therefore, from clinical and scientific perspectives, it is quite crucial to comprehensively investigate the oxidative stress biomarkers in periodontal tissues. Moreover, the exacerbation of systemic oxidative stress may represent an important mechanism for the mutual aggravation of DM and periodontitis. Furthermore, the investigation of antioxidant enzymes such as superoxide dismutase (SOD) and catalase is needed to clarify the complicated oxidative stress process. Therefore, in our study, we evaluated the serum content of malondialdehyde (MDA), a thiobarbituric acid-reactive substance used as a marker of lipid peroxidation, and serum SOD activity, to assess the possible systemic oxidative damage involved in DP.

One potential mechanism accounting for the aggravation of periodontitis by DM may be the downregulation of local antioxidant transcription factors, such as nuclear factor-E2-related factor 2 (Nrf2). Nrf2 is a key transcription factor that regulates a large group of antioxidant and detoxifying enzymes. Its activation represents a crucial cellular defense mechanism in ameliorating oxidative damage [6]. Downregulation of Nrf2 and subsequent inhibition of antioxidant production have been associated with more advanced periodontitis [7]. In addition, Nrf2 overexpression has been proved to activate antioxidative enzymes and thus effectively inhibit periodontal ligament stem cell apoptosis in the local oxidative stress microenvironment of periodontitis [8]. Moreover, Nrf2 also plays important roles in DM-related diseases [9]. Nrf2 deficiency results in increased ROS level, leading to higher blood glucose level and impaired insulin signaling in murine models [10]. Furthermore, the activation of Nrf2 signaling provides substantial therapeutic benefits on DP [9, 11]. However, it remains unclear whether Nrf2 is involved in the aggravation of periodontitis by DM.

In the present study, we hypothesized that enhanced oxidative damage and downregulation of Nrf2 contributed to the aggravated pathogenesis of periodontitis by DM. Herein, we investigated both the local and systemic oxidative damage of DP in rats. In addition, the expression of Nrf2 and its relationship with periodontal pathological indexes were analyzed to demonstrate its potential role in DP.

## 2. Materials and Methods

### 2.1. Animals

Male Wistar rats weighing 200–220 g (6–8 weeks old) were purchased from the Animal Center of Wenzhou Medical University and acclimated (ventilated controlled room at 20°C on a 12 h light/dark cycle with free access to water and food) for 1 week. All experimental protocols were approved by the Animal Ethics Committee of Wenzhou Medical University (WYKQ2015001).

### 2.2. Group Allocation

Sixty-four rats were divided randomly into four groups of sixteen each. The study groups were as follows: C—no treatment, P—experimentally induced periodontitis for 2 weeks, D—experimentally induced diabetes mellitus, and DP—experimentally induced diabetes and periodontitis. Two weeks after confirmation of diabetes, periodontitis was induced for 2 weeks.

### 2.3. Induction of Diabetes

Rats were given a single intraperitoneal injection of streptozotocin (STZ) (50 mg/kg; Sigma, St. Louis, MO, USA) to induce diabetes. Three days following STZ administration, rats with plasma glucose concentrations of >16.7 mmol/L were selected as the STZ-induced diabetes group.

### 2.4. Induction of Periodontitis

Sterile, 3–0 black braided nylon thread (Surgilon; USS/DG, Norwalk, CT, USA) was placed around the bilateral lower first molars [12]. Rats were sacrificed 2 weeks after ligation. The residual left side of the mandibles was fixed by paraformaldehyde and subsequently used for the macroscopic evaluation of alveolar bone loss.

### 2.5. Microscopic Examination of Periodontal Bone Loss

The distance from the amelocemental junction (ACJ) to the alveolar crest (AC) was measured in the left sides of the mandible as described previously [12]. The mean of the recordings for each tooth (expressed in mm) was used as a measurement of bone loss.

### 2.6. Histologic Tissue Preparation and Analysis

Gingivomucosal tissues surrounding the left side of the mandibles were fixed for the analysis of apoptosis of periodontal cells, as previously described [13]. Cryostat sections (6 *μ*m thick) were obtained. The presence of apoptotic periodontal cells in periodontal soft tissues was detected by terminal deoxynucleotidyl transferase dUTP nick end labeling (TUNEL) kits from Roche (Mannheim, Germany).

The right sides of the mandible were fixed in paraformaldehyde for 48 hours, decalcified in ethylenediamine tetraacetic acid (EDTA), and embedded in paraffin. Serial slices (4 *μ*m thick) were prepared in the mesiodistal plane and used for histopathologic and immunohistochemical analyses.

### 2.7. Immunohistochemistry

For immunohistochemistry, the sections were stained with anti-4-HNE (1 : 400), anti-3-NT (1 : 400), and anti-8-OHdG (1 : 200) from Abcam Biotechnology and anti-Nrf2 (1 : 400) from Santa Cruz Biotechnology. The color was developed with 3-3′-diaminobentizine tetrahydrochloride, and sections were counterstained with Mayer's hematoxylin. Three regions of interest were defined for each site (ligature and control): alveolar bone crest, sulcular epithelium, and gingival connective tissue. The number of positive cells and total cells per unit area (0.01 × 0.01 mm) was determined in the periodontal ligament at a magnification of 400x. The ratios of each substance-positive cell to total cells were calculated.

### 2.8. Western Blot Analysis

Frozen, powdered samples of the gingiva were solubilized in radioimmunoprecipitation assay (RIPA) buffer (50 mM Tris-HCl, pH 8.0, with 150 mM sodium chloride, 1.0% IGEPAL CA-630 (NP-40), 0.5% sodium deoxycholate, and 0.1% sodium dodecyl sulfate (Sigma, St. Louis, MO, USA)). The lysates were assayed by Western blot using antibodies to Nrf2 (1 : 1000; Santa Cruz) and glyceraldehyde-3-phosphate dehydrogenase (GAPDH 1 : 10000; Abcam). The protein bands were detected using the Bio-Rad imaging system (Bio-Rad, Hercules, CA, USA) and quantified using NIH ImageJ software. The expression of protein bands was corrected according to GAPDH density.

### 2.9. Real-Time Polymerase Chain Reaction Analysis of Nrf2 mRNA Expression

For messenger RNA (mRNA) expression analysis, total RNA was extracted from the excised tissues using TRIzol reagent (Invitrogen, Carlsbad, CA, USA) and quantified by measuring the absorbance at 260 and 280 nm. cDNA was amplified using gene-specific primers: Nrf2: NM_031789.2 forward, TGTCAGCTACTCCCAGGTTG; reverse, ATCAGGGGTGGTGAAGACTG. GAPDH: NM_002046.5 forward, ACAGTCAGCCGCATCTTCTT; reverse, ACGACCAAATCCGTTGACTC. Real-time polymerase chain reaction (RT-PCR) analysis was then performed for 30 cycles of 1 minute each at 94°C (denaturation), 60°C (annealing), and 72°C (elongation), and final extension was performed at 72°C for 10 minutes.

### 2.10. Blood Sample Collection and Analyses of Serum Samples

Blood sample (5 mL) was collected from the heart of each rat by using 5 mL vacutainer tubes containing 10 mg of EDTA before the rats were euthanized by an excess of anesthesia. The blood samples were centrifuged to obtain serum, which was stored in the dark at 20°C. The level of MDA was determined by the lipid peroxidation MDA Assay Kit (Beyotime Biotechnology, China). In brief, the obtained serum of each group was subjected to the measurement of MDA level according to the manufacturer's protocol. MDA reacts with thiobarbituric acid (TBA) to generate an MDA-TBA adduct. The MDA-TBA adduct was then quantified colorimetrically at 530 to 540 nm absorbance.

The activity of total SOD was detected using the Total Superoxide Dismutase Assay Kit with WST (water-soluble tetrazolium salt) (Beyotime Biotechnology, China). The kit uses WST-1 that produces a water-soluble formazan dye upon reduction with superoxide anion. The rate of the reduction with a superoxide anion is linearly related to the activity of xanthine oxidase (XO) and is inhibited by SOD. The samples were scanned with a spectrophotometer at the absorbance of 450 nm. The rate of change in absorbance was converted to units of enzyme activity, determined from a standard curve. We used the BCA protein assay kit (Thermo Fisher Scientific Inc., Waltham, MA, USA) to quantify protein concentration of each sample. Enzyme activity was then standardized to milligram protein.

### 2.11. Statistical Analysis

Data are expressed as mean ± SD. Statistics were analyzed using StatView software (SAS Institute). For comparisons between multiple groups, one-way ANOVA was used followed by individual post hoc Fisher tests when applicable. Finally, multiple linear regression analysis was used in our study. *p* < 0.05 was considered to be statistically significant.

## 3. Results

### 3.1. Blood Glucose Level and Body Weight

**Figure 1:**
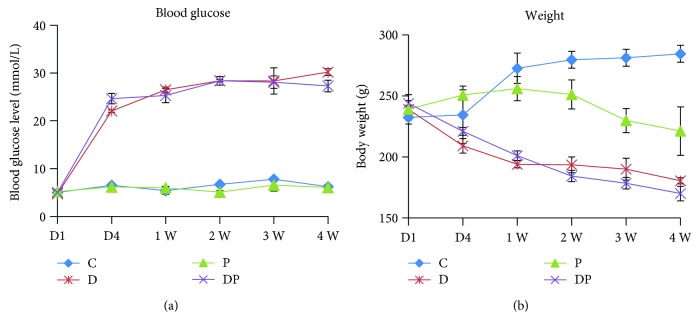
Nonfasting glucose levels (mmol/L) and body weight (g) at various time points in each group. Data are presented as mean ± SD (*n* = 16/each group). D: day; W: week.

Both the DP and D groups exhibited hyperglycemia, resulting in a significantly higher nonfasting blood glucose level than that in the P and C groups (Figure 1(a)). At the end of the experiment, rats in the DP group showed the lowest body weight, followed by those in the D and P groups (Figure 1(b)).

### 3.2. Alveolar Bone Loss and Apoptosis of Periodontal Cells

**Figure 2:**
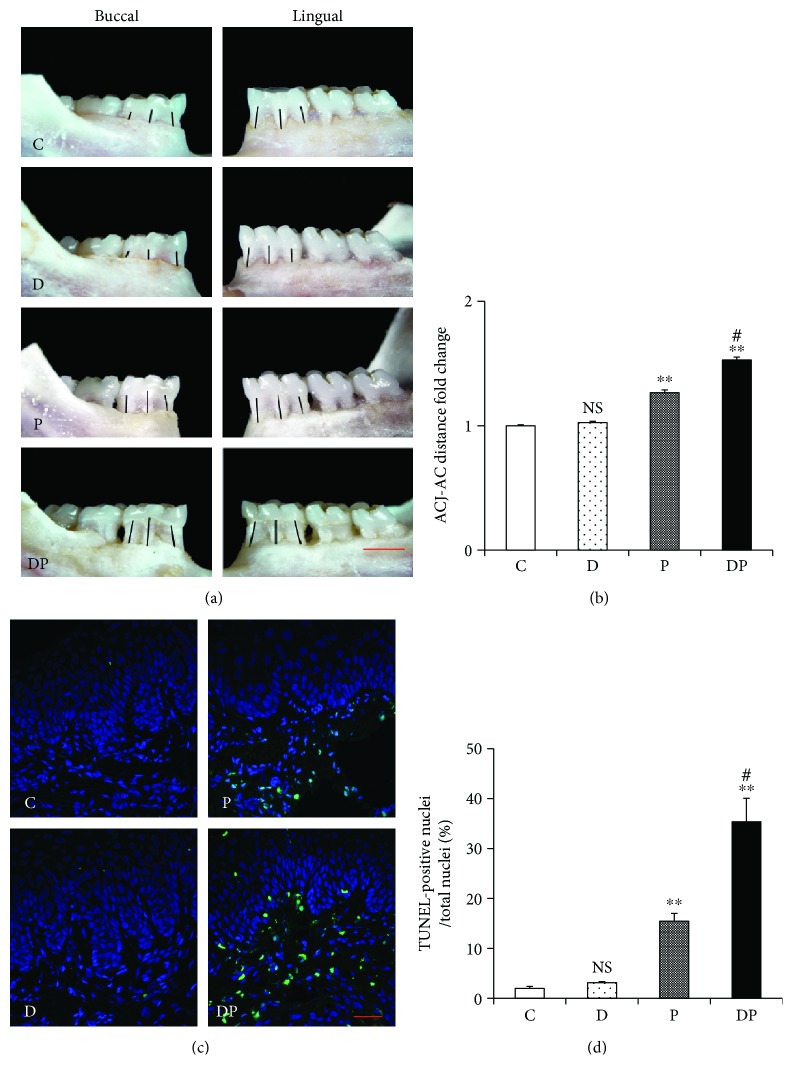
Diabetes aggravated alveolar bone loss and apoptosis of periodontal cells in periodontitis. (a) Macroscopic aspects of the mandibles observed in the C, D, P, and DP groups. The black lines showed the distance from the amelocemental junction (ACJ) to the alveolar crest (AC) (bar = 1 mm). (b) Quantitative analysis of the ACJ-AC distance and bone loss area. (c) Apoptosis of periodontal cells was determined by the TUNEL assay and expressed as the apoptotic rate. TUNEL staining (with DAPI costaining) with nuclei being stained with blue is shown. TUNEL-positive nuclei are green/cyan (bar = 25.5 um). (d) Demonstrated apoptotic cell number per microscopic field. Data shown are mean ± SD (*n* = 6/each group). NS: nonsignificantly different from the C group. ^∗∗^*p* < 0.01 versus the C group. ^#^*p* < 0.05 versus the P group. (C: no treatment; P: experimentally induced periodontitis; D: experimentally induced diabetes mellitus; DP: experimentally induced diabetes and periodontitis.)

Rats in the DP group showed the highest rate of bone loss, which was significantly higher than that in the P group. In addition, rats from the P group showed more bone loss than those in the C group. Bone loss was slightly increased in the D group compared with that in the C group; however, no significant difference was found (Figures 2(a) and 2(b)).

Apoptotic periodontal cells were found in both DP and P groups, but were rarely seen in the D and C groups. The number of apoptotic periodontal cells in the DP group was significantly higher than that in the P group. In addition, the P group showed a significantly higher level of apoptosis than the C group. However, no significant difference in the level of apoptosis was observed between the D and C groups (Figures 2(c) and 2(d)).

### 3.3. Expression Levels of Local and Systemic Oxidative Stress Biomarkers

**Figure 3:**
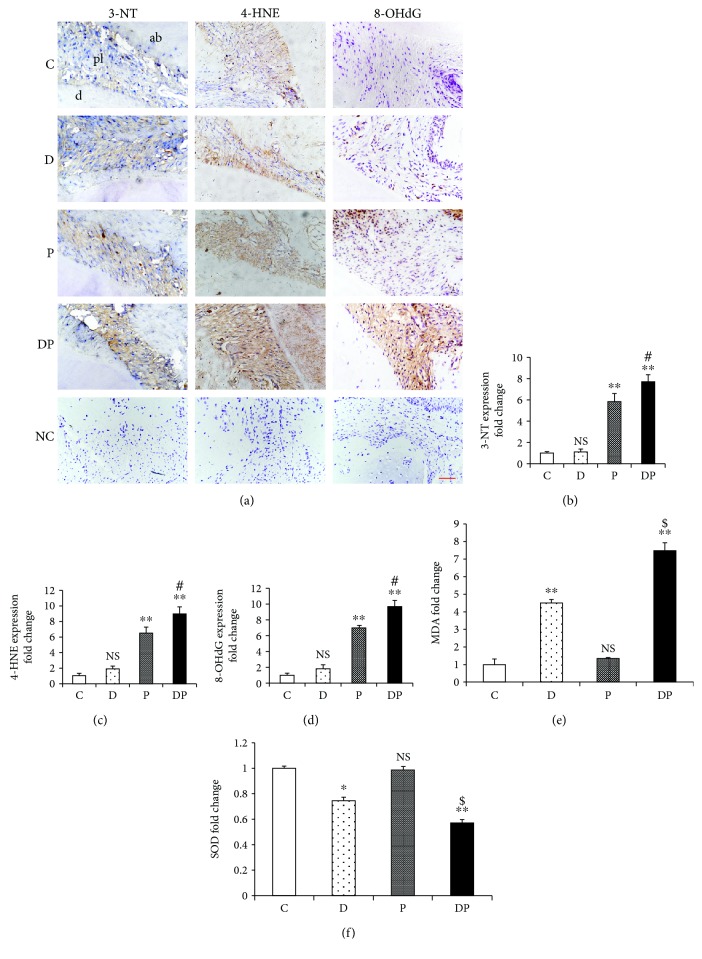
Diabetes enhanced the protein expression levels of 3-NT, 4-HNE, and 8-OHdG in ligature-induced periodontal lesions. (a) Representative figures from anti-3-NT, 4-HNE, and 8-OHdG immunohistochemical staining of alveolar sections from the C, D, P, and DP groups (NC: negative control). (b–d) Quantitative analysis of 3-NT, 4-HNE, and 8-OHdG expression in the periodontal area. Scale bar = 100 *μ*m; original magnification ×400. Diabetes increased the serum MDA level and decreased serum SOD activity in periodontitis. Quantitative analysis of MDA expression (e) and SOD activity (f) among the indicated groups. NS: nonsignificantly different from the C group; ^∗∗^*p* < 0.01: significantly different from the C group; ^$^*p* < 0.01 versus the D group. (C: no treatment; P: experimentally induced periodontitis; D: experimentally induced diabetes mellitus; DP: experimentally induced diabetes and periodontitis.)

Quantitative analysis showed that rats from the DP group showed significantly elevated levels of 3-NT-, 4-HNE-, and 8-OHdG-positive cells, which resulted in a 32%, 38%, and 38% increase, respectively, compared with those from the P group. Furthermore, the P group showed increased levels of 3-NT, 4-HNE, and 8-OHdG compared with the C group. However, no significant difference was observed between the D and C groups for these biomarkers (Figures 3(a)–3(d)).

The serum level of MDA was markedly higher in rats from the DP group than in those from the other groups. Conversely, the serum SOD activity of the DP group was decreased by 42%, 23%, and 43% when compared with that of the P, D, and C groups, respectively. When compared with the C group, the D group presented significantly increased expression of MDA and reduced SOD activity. However, there was no significant difference in serum MDA level or SOD activity between the P and C groups (Figures 3(e) and 3(f)).

### 3.4. Protein and mRNA Expression of Nrf2 in Periodontal Tissues

**Figure 4:**
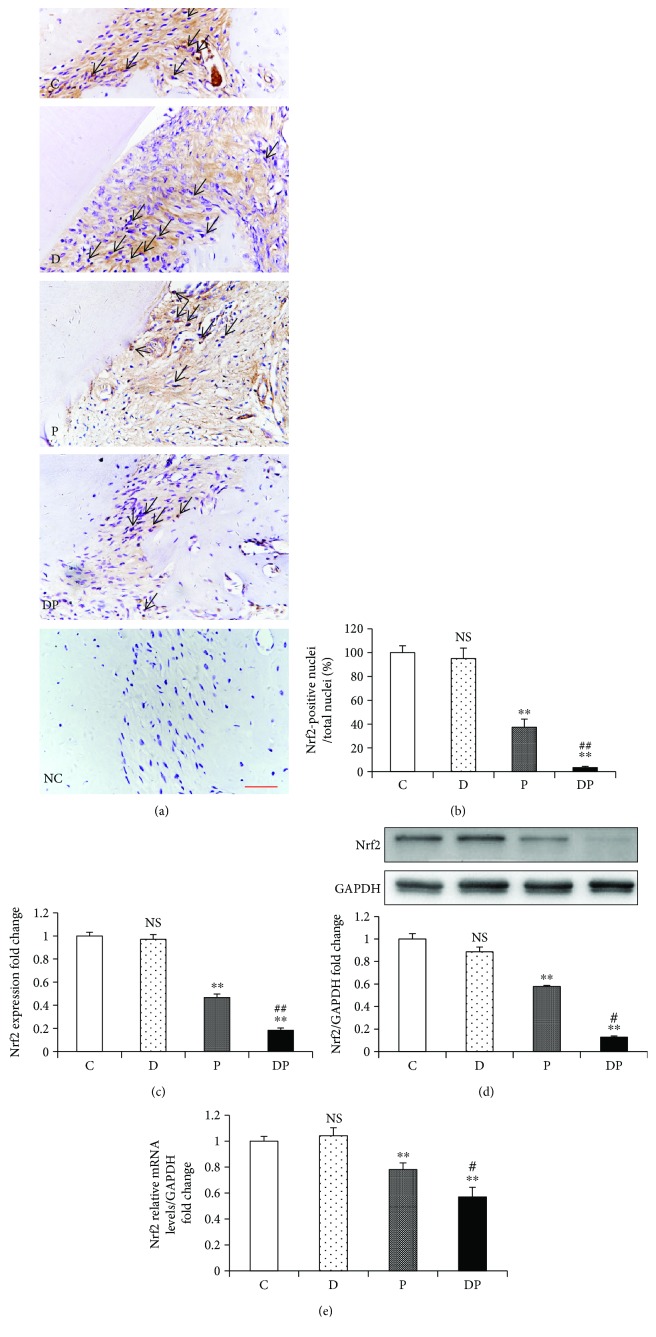
Diabetes exacerbated the decrease in the protein level and gene expression of Nrf2 in periodontitis. (a) Representative figures from anti-Nrf2 immunohistochemistry staining of alveolar sections from the C, D, P, and DP groups (NC: negative control). Quantitative analyses of (b) Nrf2-positive nuclei and (c) the expression level in the gingiva. (d) Representative immunoreactive bands for Nrf2 in periodontal tissues in the indicated groups and the total protein level of Nrf2 relative to GAPDH. (e) Real-time PCR analysis of Nrf2 in periodontal soft tissues of the indicated groups. Results are expressed as mean ± SD (*n* = 6). NS: nonsignificantly different from the C group; ^∗∗^*p* < 0.01 significantly different from the C group; ^#^*p* < 0.05 and ^##^*p* < 0.01 versus the P group. (C: no treatment; P: experimentally induced periodontitis; D: experimentally induced diabetes mellitus; DP: experimentally induced diabetes and periodontitis.)

The immunohistochemical staining demonstrated reduced protein expression of Nrf2 in both the DP and P groups in the nuclei and cytoplasm compared with that in the C group. Furthermore, rats from the DP group displayed significantly less Nrf2-positive cells than rats from the P group (Figures 4(a)–4(c)). Western blot analysis further confirmed that Nrf2 protein expression was significantly reduced in rats from the DP group compared with that in rats from the other groups (Figure 4(d)). When compared with the C group, Nrf2 mRNA expression level was significantly reduced in the DP and P groups. Moreover, the DP group showed a further decrease in Nrf2 expression compared with the P group (Figure 4(e)).

### 3.5. Multiple Linear Regression Analysis

Alveolar bone loss was significantly associated with the alterations in the levels of 3-NT, 4-HNE, 8-OHdG, M5DA, SOD, and Nrf2 in the DP and D groups (3-NT: correlation coefficient: *r* = 0.046 (*p* < 0.0001), 4-HNE: *r* = 0.03 (*p* = 0.002), 8-OHdG: *r* = 0.123 (*p* = 0.006), MDA: *r* = 0.084 (*p* = 0.002), SOD: *r* = −0.877 (*p* = 0.009), and Nrf2: *r* = −0.984 (*p* = 0.049)) (Table 1).

Apoptosis of periodontium cells was also closely related to the alterations in the levels of 3-NT, 4-HNE, 8-OHdG, MDA, SOD, and Nrf2 in the DP and D groups (3-NT: *r* = 0.034 (*p* < 0.0001), 4-HNE: *r* = 0.022 (*p* = 0.001), 8-OHdG: *r* = 0.087 (*p* = 0.009), MDA: *r* = 0.034 (*p* = 0.002), SOD: *r* = −0.618 (*p* = 0.015), and Nrf2: *r* = −0.781 (*p* = 0.026)) (Table 2).

The alteration in the level of Nrf2 was strongly negatively correlated to changes in the levels of 3-NT, 4-HNE, 8-OHdG, MDA, and SOD in the DP and D groups (3-NT: *r* = −0.026 (*p* = 0.038), 4-HNE: *r* = −0.022 (*p* = 0.002), 8-OHdG: *r* = −0.079 (*p* = 0.024), MDA: *r* = −0.034 (*p* = 0.001), and SOD: *r* = 0.719 (*p* < 0.0001)) (Table 3).

Among all the oxidative stress biomarkers, according to the correlation coefficient, SOD showed the strongest correlation with periodontal destruction (*r* = −0.877, −0.618, *p* = 0.009, 0.015) and the expression of Nrf2 (*r* = 0.719, *p* < 0.0001), whereas for local oxidative stress biomarkers the parameter was 8-OHdG (*r* = 0.123, 0.087, *p* = 0.006, 0.009) (*r* = −0.079, *p* = 0.024).

## 4. Discussion

The high prevalence and severity of periodontitis in diabetic populations have become a major worldwide health problem. Thus, there is an urgent need to clarify the mechanism of the pathogenesis of DP. The mechanism by which DM exerts its deleterious effects on periodontitis is multifactorial and most likely associated with oxidative stress [14, 15]. In the present study, we, for the first time, provided substantial evidence that both local and systemic oxidative damage and Nrf2 downregulation are involved in the aggravation of periodontitis by DM.

Consistent with our previous study [16], both type 1 DM and a periodontitis model were successfully established in rats and characterized by hyperglycemia, significant alveolar bone loss, and enhanced apoptosis of periodontal cells. Furthermore, the microscopic and histologic changes were consistent with our previously published data [16], confirming that DM enhances periodontal destruction.

Our study focused on both local and systemic oxidative stress status under periodontitis and/or DM conditions. We found that under the condition of periodontitis, the local periodontal oxidative stress was significantly enhanced, whereas the systemic oxidative stress level was not affected. The serum MDA level and SOD activity of the P and C groups did not reach statistical significance, which may be due to the tissue-preserving antioxidant-adaptive mechanism against the oxidative stress developed in periodontitis [17]. Furthermore, our study showed that although systemic oxidative stress was aggravated in diabetic rats, no significant difference in oxidative stress level was detected in the local periodontal tissues between the D and C groups. Previous animal studies have demonstrated that when DM is induced in rats for 3, 6, 9, and 12 months, marked alveolar bone loss is observed in periodontal tissues [18, 19]; thus, we inferred that the 1-month duration in our study might not be long enough for DM to affect either bone loss or periodontal oxidative stress level in rats without periodontitis. Regarding DP, both local and systemic oxidative stress levels were enhanced, as reflected by higher expression of 3-NT, 4-HNE, and 8-OHdG in periodontal tissues, accompanied by elevated serum level of MDA and decreased serum SOD activity. On the basis of these data, DM aggravated both the local and systemic oxidative damage in periodontitis. To our knowledge, the present study is the first to comprehensively report that oxidative protein damage, lipid peroxidation, and DNA oxidation in periodontal tissues are closely related to the periodontal destruction in DP.

Proteins are major targets for oxidative damage [20]. Nitrotyrosine represents a commonly used biomarker for protein oxidation and is expressed in various tissues of diabetic patients [21, 22]. Our study showed that the expression of 3-NT protein was significantly elevated in the periodontal tissues of rats with DP. The results of multiple regression analysis further showed that the increased 3-NT expression was closely related to alveolar bone loss and apoptosis of periodontium cells. Increased nitrotyrosine formation has been proved to be associated with various tissue injuries in lipopolysaccharide- (LPS-) treated rats, such as aortic and brain damage [23, 24]. LPS from *Porphyromonas gingivalis*, one of the most important pathogenic bacteria of periodontitis, contributes to the destruction of periodontal tissues. Given the alteration of 3-NT in DP, we inferred that nitrotyrosine might be a crucial factor that links LPS-mediated periodontitis and systemic diseases, such as DM.

Oxidative stress also leads to lipid peroxidation and results in the production of aldehydes such as 4-HNE [25]. 4-HNE levels in gingival crevicular fluid (GCF) [26] and serum [27] have been used as oxidative biomarkers to detect the local and systemic impact of chronic periodontitis. Our results showed that the 4-HNE level was the highest in the periodontal tissues of rats in the DP group, which suggested that lipid peroxidation in periodontitis is greatly enhanced by DM. Furthermore, 4-HNE has been suggested to impair mitochondrial function and energy production, as indicated by a significant decrease in mitochondrial membrane potential, mitochondrial oxygen consumption, and depletion of the reserve capacity [28]. Our previous studies showed that mitochondrial oxidative stress and dysfunction contributed largely to the DM-aggravated periodontal tissue damage. Thus, the 4-HNE-mediated mitochondrial abnormalities might be a crucial mechanism underlying the progression of DP.

DNA oxidative damage is involved in a variety of diseases, including periodontitis [29] and DM [30]. 8-OHdG represents the most widely accepted assay for oxidative DNA damage [31]. Higher 8-OHdG level has been found in patients with periodontitis [32]. DM is also linked to oxidative DNA damage and decreased efficacy of DNA repair [30]. Therefore, oxidative DNA damage represents a shared pathology of periodontitis and DM. However, thus far, DNA oxidative damage has not been investigated in DP. In our study, significantly higher 8-OHdG level was detected in the periodontal tissues of rats with DP, which indicated that DM aggravated DNA oxidation in periodontitis. Furthermore, the increased 8-OHdG expression was associated with the enhanced alveolar bone loss and periodontal ligament cell apoptosis involved in DP. Mitochondria are a major source for and also the principal attack target of ROS [33]. Mitochondrial DNA (mtDNA) damage is more extensive and persists longer than nuclear DNA damage following oxidative stress [34]. A few studies showed that the salivary 8-OHdG level might signify premature oxidative mtDNA damage in diseased gingival tissues [35]. Our previous studies demonstrated that DM reduced the mtDNA copy number in periodontitis, suggesting enhanced mtDNA impairment in DP [16]. Whether the 8-OHdG level in periodontal tissues can signify mtDNA damage in DP needs further investigation.

With regard to systemic oxidative stress biomarkers, our results showed that the MDA level was significantly elevated, whereas SOD activity was decreased, in the serum of rats in the DP group compared with that in the other groups. It is interesting that SOD, among all the oxidative stress biomarkers, showed the strongest correlation with periodontal destruction. In the previous studies, substantial data were available in comparing oxidative stress markers in patients with DP or periodontitis [17, 36, 37]. However, the results were inconsistent and showed significantly increased or no changes in the level of MDA and SOD activity [17, 37]. This inconsistency can be attributed to factors such as time-dependent changes in the enzyme activity, enzyme resisting oxidative stress by adaptation, severity of the diseases, and the differences in tested tissues. Our results may contribute more evidence to the role of systemic oxidative stress in DP and showed some novel directions that deserve further investigation.

It is well established that Nrf2 is responsible for cellular defense against oxidative stress by inducing hundreds of antioxidant and detoxifying enzymes [6]. In response to oxidative stress, cytoplasmic Nrf2 is released from Kelch-like ECH-associated protein 1 and binds to antioxidant response elements in the promoter region of many antioxidant enzymes [38]. There is abundant evidence that Nrf2 plays a central role in protection against periodontal tissue destruction [39, 40]. Compared with the control mice, Nrf2 knockout mice exhibited aggravated oxidative damage and enhanced alveolar bone and attachment loss at sites with chronic periodontitis [7]. Furthermore, Nrf2 plays an essential role in the progression of diabetic complications and is capable of preventing DM-induced oxidative stress [9, 41]. However, the role of Nrf2 in the pathogenesis of DP remains unclear. Our study demonstrated that both the mRNA and protein levels of Nrf2 in the nuclei and cytoplasm were reduced in DP. In addition, the downregulation of Nrf2 was closely related with the enhanced local and systemic oxidative damage and aggravated periodontal damage in diabetic rats with periodontitis. Furthermore, Nrf2 is a prominent player in supporting the structural and functional integrity of the mitochondria. Following oxidative stress, Nrf2 affects the mitochondrial ROS production, mitochondrial membrane potential, activity of mitochondrial electron transport chain complex I, and mitochondrial biogenesis [42]. Our previous study showed that rats with DP presented more severe mitochondrial dysfunction than those with periodontitis alone [16]. On the basis of these data, the downregulation of Nrf2 might be strongly linked to the mitochondrial abnormalities involved in DP. Previously, Nrf2 upregulation has been proved to prevent periodontitis and DM-related oxidative damage [40, 41]. Therefore, the application of Nrf2 activator may be a viable treatment option to prevent the oxidative damage, mitochondrial dysfunction, and subsequent tissue destruction in DP.

Several antioxidant compounds such as curcumin, sulforaphane, and resveratrol have already been shown to promote the expression and activation of Nrf2 [43]. The pretreatment of curcumin induces the activation of Nrf2 and an antioxidant response and prevents hemin-induced neuronal death of rats [44]. Sulforaphane prevents angiotensin II-induced cardiomyopathy partially through the Akt/GSK-3*β*/Fyn pathway-mediated upregulation and activation of Nrf2 [45]. Sulforaphane also prevents diabetic nephropathy through upregulation of Nrf2 [46]. Similarly, resveratrol attenuates testicular apoptosis in type 1 diabetic mice through the upregulation of Nrf2 expression and function by Akt-mediated Nrf2 activation and p62-dependent Keap1 degradation [41]. Nrf2/antioxidant defense pathway activated by resveratrol was further found to be involved in preventing the progression of ligature-induced periodontitis [40]. However, to the best of our knowledge, there has been no report demonstrating the application of the abovementioned antioxidants to treat DP. On the other hand, several other bioactive agents that can activate Nrf2, such as melatonin, conjugated linoleic acid, and boric acid [47–49], have also been shown to suppress DP in other studies [50–52]. Further studies should be performed to provide direct evidences for the involvement of Nrf2 in the therapeutic effect of antioxidants on DP.

The present study also had limitations. First, the exact role of Nrf2 and its possible downstream effectors in DP remain elusive. In future studies, we will use an activator or inhibitor of Nrf2 and the Nrf2 knockout mice model to provide more information. Second, more studies are needed to further investigate the local and systemic oxidative damage and to determine the precise role of Nrf2 in patients with DP.

## 5. Conclusion

Our study offered new insights into the role of enhanced local and systemic oxidative damage and Nrf2 downregulation in periodontal tissue destruction aggravated by DM. The inhibition of oxidative stress and regulation of Nrf2-dependent antioxidants may represent novel therapeutic strategies for DP.

## Figures and Tables

**Table 1:** Multiple linear regression analysis between alveolar bone loss, oxidative stress biomarkers, and Nrf2 in diabetic periodontitis and diabetes mellitus groups.

Independent variables	Dependent variable ACJ-AC distance (mm)				
	Coef.	Std. err.	*p* value	95% CI	
Constant	1.279	0.059	<0.0001	1.135	1.424
3-NT	0.046	0.007	<0.0001	0.030	0.062
4-HNE	0.03	0.006	0.002	0.016	0.045
8-OHdG	0.123	0.029	0.006	0.051	0.194
MDA	0.084	0.014	0.002	0.047	0.121
SOD	−0.877	0.23	0.009	−1.438	−0.315
Nrf2	−0.984	0.399	0.049	−1.959	−0.008

*p* < 0.0001; *R*^2^ = 0.889.

**Table 2:** Multiple linear regression analysis between apoptosis of periodontium cells, oxidative stress biomarkers, and Nrf2 in diabetic periodontitis and diabetes mellitus groups.

Independent variables	Dependent variable apoptosis of periodontium cells (%)				
	Coef.	Std. err.	*p* value	95% CI	
Constant	0.103	0.034	0.024	0.019	0.188
3-NT	0.034	0.005	<0.0001	0.023	0.045
4-HNE	0.022	0.004	0.001	0.013	0.032
8-OHdG	0.087	0.023	0.009	0.031	0.143
MDA	0.034	0.006	0.002	0.019	0.05
SOD	−0.618	0.183	0.015	−1.065	−0.17
Nrf2	−0.781	0.265	0.026	−1.429	−0.134

*p* < 0.001; *R*^2^ = 0.838.

**Table 3:** Multiple linear regression analysis between Nrf2 expression and oxidative stress biomarkers in diabetic periodontitis and diabetes mellitus groups.

Independent variables	Dependent variable Nrf2 expression				
	Coef.	Std. err.	*p* value	95% CI	
Constant	0.413	0.047	0.013	0.297	0.528
3-NT	−0.026	0.01	0.038	−0.05	−0.002
4-HNE	−0.022	0.004	0.002	−0.032	−0.011
8-OHdG	−0.079	0.026	0.024	−0.144	−0.015
MDA	−0.034	0.006	0.001	−0.049	0.019
SOD	0.719	0.089	<0.0001	0.501	0.937

*p* < 0.0001; *R*^2^ = 0.916.

## Data Availability

The data used to support the findings of this study are available from the corresponding author upon request.
